# Regular Exercise Modifies Histopathological Outcomes of Pharmacological Treatment in Experimental Autoimmune Encephalomyelitis

**DOI:** 10.3389/fneur.2018.00950

**Published:** 2018-11-20

**Authors:** Danielle Bernardes, Alexandre Leite Rodrigues de Oliveira

**Affiliations:** Laboratory of Nerve Regeneration, Department of Structural and Functional Biology, Institute of Biology, University of Campinas, Campinas, Brazil

**Keywords:** experimental autoimmune encephalomyelitis (EAE), treadmill exercise, disease modifying therapies (DMTs), glatiramer acetate (GA), dimethyl fumarate (DMF)

## Abstract

**Background:** Although it has been suggested that healthier lifestyle may optimize effects of the immunomodulation drugs for treating multiple sclerosis (MS), the knowledge regarding this kind of interactions is limited.

**Objective:** The aim of the present study was to investigate the effects of treadmill exercise in combination with pharmacological treatment in an animal model for MS.

**Methods:** C57BL/6J female mice were subjected to daily treadmill exercise for 4 weeks before immunization and 6 weeks before clinical presentation of disease. Dimethyl fumarate (DMF) or glatiramer acetate (GA) were administered after the first clinical relapse. Histopathological analyses were carried out in the lumbar spinal cord at peak disease and at 1 or 14 days post-treatment (dpt).

**Results:** Exercised-GA treated animals demonstrated decreased astrocytic response in the spinal dorsal horn with an improvement in the paw print pressure. Exercised-DMF treated animals showed an increased microglial/macrophage response on both ventral and dorsal horn that were associated with clinical improvement and synaptic motoneuron inputs density.

**Conclusion:** The present data suggest that prior regular exercise can modify the effects of pharmacological treatment administered after the first relapse in a murine model for MS.

## Introduction

Among the Disease Modifying Therapies (DMTs) used nowadays aiming at diminishing relapses and improving quality of life for Multiple Sclerosis (MS) patients, there are two major groups of drugs referred as immunomodulatory or immunosuppressive lines (1). The rationale for changing treatment to immunosuppressive therapies arise from the insufficient reduction of relapses by the immunomodulation strategy (1, 2). However, there are evidences in favor of optimizing immunomodulatory response by using behavioral strategies that could enhance the effects of the immunomodulatory DMTs (2, 3). Thus, prior physical fitness may affect the response to DMTs in MS patients yielding important beneficial response to the DMTs.

Glatiramer acetate (GA) has been used as a DMT for more than 20 years. The most accepted mechanism of action for GA is a switch from Th1 to a Th2 response that is anti-inflammatory (1). The alteration of CNS infiltrated T cells leads to decreased glial reactivity and white matter demyelinating lesions with stimulation of remyelination and even protection against the neuronal damage (4). In the same way, the newly approved dimethyl fumarate (DMF) is considered as a DMT for MS because of its basic mechanism of action related to a switch response from Th1 to Th2 (5). In fact, three different clinical studies have demonstrated significant improvements of relapses rate and decreased lesions in magnetic resonance imaging (MRI) for most of MS patients and, therefore, DMF has been considered a double effect drug with both immunomodulatory and neuroprotective roles (6).

Nowadays, physical exercise has been considered an important option to improve quality of life of MS patients (7). In fact, there is evidence about fitness level and the number of MRI lesions in MS patients (8). More importantly, it appears as low levels of fitness constitutes a risk factor for developing MS (9). Besides the obvious cardiorespiratory benefits, physical fitness has an important and positive impact on both immune and nervous systems (10).

One strategy to address the interaction between prior exercise and DMT treatment is using an animal model such as the experimental autoimmune encephalomyelitis (EAE). EAE presents histopathological hallmarks that outline the human disease and allows deep investigation of morphological alterations associated with different options of treatment (11). Besides, clinical presentation as well as associated motor and sensory disturbances may be under investigation to test the efficiency of some therapeutic strategies. In fact, both GA and DMF, as well as physical exercise alone, have been investigated in EAE mice providing insights regarding therapy mechanisms. However, there are no studies associating GA and DMF in EAE mice that have been subjected to physical exercise. Such combined treatment may provide useful information regarding better understanding the pathogenesis of MS as well improving the efficiency of DMTs.

Therefore, the aim of the present study was to investigate the effectiveness of physical exercise for optimizing two different DMTs in the EAE model. For that, we used 6 weeks of treadmill exercise prior to the onset of the disease and the animals were treated with GA or DMF after the first relapse post exacerbation.

## Methods

### Ethical approval and animal conditions

Experiments were carried out in accordance to the international guidelines and principles regulated by the National Council of Animal Experimentation for the care and use of animals (CONCEA, Brazil). After the ethics committee approval (3844-1/2015), the Multidisciplinary Center for Biological Research (CEMIB/UNICAMP, Campinas, SP, Brazil) supplied the 96 female C57BL/6J mice (4–6 weeks old) used in the present study. All animals were maintained in standard conditions in the animal house of the Laboratory of Nerve Regeneration on a 12/12 h light/dark cycle and, were provided with food and water *ad libitum*.

### Study design

After a week of environmental adaptation, mice were randomly assigned to the no-exercise (*n* = 45) and exercised (*n* = 46) groups. At the beginning of the fifth week, all the animals were induced for the experimental autoimmune encephalomyelitis (EAE) protocol and the exercise paradigm was maintained until 10 dpi (days post induction), completing 6 weeks of regular exercise. The clinical signs developed on 11–12 dpi and peaked at around 16–17 dpi, when 7 and 8, respectively no-exercise and exercised mice, were perfused to identify peak disease morphological alterations. From the 21st dpi (clinical signs remission) some of the reminiscent mice (12 no-exercise and 14 exercised) were treated with GA (EAE-GA and EAE-Ex-GA groups). Another set of mice (14 no-exercise and 12 exercised) were treated with DMF (EAE-DMF and EAE-Ex-DMF groups). Finally, a group of animals (12 no-exercise and 12 exercised) was just followed as control groups of the drug treatments (EAE and EAE-Ex groups). Weight, clinical score, gait parameters and, the motor behavior of all animals were collected as described below. To identify morphological alterations of the exercise protocol associated with the two drug treatments, the animals were perfused at 1 or 14 days post-treatment (dpt). The untreated animals (EAE and EAE-Ex groups) were perfused at 28 or 42 days post induction and an additional group of 5 animals was perfused in naïve condition.

### Physical exercise protocol

In order to standardize the training outcome (evaluation by catwalk gait analysis and motor rotarod), we chose to investigate the effects of treadmill exercise on the development of EAE (12). For that, mice were first familiarized to the motorized treadmill for 5 consecutive days with a progressive increase of training time (5 to 25 min), a speed of 6 m/min and 11° of inclination. Next, the exercise protocol consisted of five more weeks (5 days/week) of forced running at 11 m/min, one session/day for 30 min. These parameters of intensity and duration of the exercise were chosen because it has been previously found to promote neuroprotection in rats (13) and mice (14). To account the stress associated with the environment, the no-exercise group of mice was placed on a bench on the side of the treadmill.

### EAE induction and clinical assessment

During the fifth week of the physical exercise protocol, EAE was induced by subcutaneous immunization (in the tail base) with an emulsion containing 100 μg of MOG_35−55_ peptide in complete Freund's adjuvant (CFA), supplemented with 4 mg/ml *Mycobacterium tuberculosis* H37Ra (Difco Laboratories, Detroit, MI, USA). *Bordetella pertussis* toxin (300 ng/animal; Sigma-Aldrich, St. Louis, MO, USA) was injected i.p. on the day of immunization and after 48 h. The body weight and the clinical score were monitored daily. Clinical evaluation was defined as follows: 0 no clinical signs, 1 tail paralysis (or loss of tail tone), 2 tail paralysis, and hind-limb weakness (visible paresis) and 3 one or two hind-limb paralysis. The referred protocol was performed as previously described (12, 15).

### Drug treatments

Since the general aim was to verify the efficiency of the exercise in optimizing the effectiveness of the two DMTs when the disease has already been installed, we use the late therapeutic method as a treatment regimen. Therefore, from the 21st dpi, mice were treated with glatiramer acetate (EAE-GA and EAE-Ex-GA groups); dimethyl fumarate (EAE-DMF and EAE-Ex-DMF groups) or were just followed as control groups (EAE and EAE-Ex groups). For GA treatment, animals received a subcutaneous injection at a dose of 0.5 mg/animal/day for 7 days (16, 17) and DMF was administered by gavage (diluted in 0.08% methylcellulose solution) at a dose of 15 mg/kg for 14 days (18).

### Gait analysis

The CatWalk system software version XT 10.1 (Noldus Inc, The Netherlands) was used to analyze the gait profile in the groups EAE, EAE-Ex, EAE-GA, EAE-Ex-GA, EAE-DMF, and EAE-Ex-DMF groups from a basal time point (before induction) to 42 days post induction (dpi). For the collection of the data, each animal was placed into the walkway and was allowed to move freely in both directions with a run duration between 0.50 and 5.00 s and a maximum allowed speed variation of 60%. A high-speed camera carried out data acquisition and the software automatically classified the paw prints. The camera gain was set to 25.01 and the detection threshold to 0.25 and the collected data were first normalized against basal values, which were settled as 100%. Four compliant runs were acquired per trial and no food restriction or reward was used. As we have demonstrated before, 19 dpi can be identified as the starting point of the walking dysfunction with a significant alteration in several gait parameters including the hind paws “mean intensity of the 15 most intense pixels” (12). This parameter is a refined measurement of the intensity of the pressure applied during a paw stance which is not recovered with the disease progression and may suggest a development of chronic mechanical allodynia (19, 20).

### Rotarod

The rotarod motor test was collected as described elsewhere (12). Briefly, there was an adaptation period of 3 days in which each mouse was placed three times on the turning wheel for 5 min. A period of 20–30 min of rest between the trials was allowed and the rotations per minute (rpm) was gradually increased (5, 10, and 16). Afterward, the animals were tested for determination of the basal condition or disease status evaluation with an acceleration parameter from 5 to 25 rpm for 6 min (360 s). The test was finalized when the animal did not maintain itself on the turning wheel voluntarily. With the disease progression, all EAE mice decrease the latency to fall which is not recovered to the basal condition for untreated mice.

### Tissue preparation

To investigate the efficacy of the association (exercise and DMTs) on the progression of the spinal cord injuries associated with the model, tissue processing was performed as described elsewhere (21). The lumbar spinal cord was selected once it is the anatomical locus of the afferent and efferent components of the sciatic nerve which has been classically associated with gait analysis. Therefore, on the specified time points (see section Study Design), all animals were anesthetized by a mixture of xylazine (80 mg/kg) and ketamine (400 mg/kg) and perfused with phosphate-buffered saline (PBS, pH 7.38) followed by buffered 4% paraformaldehyde solution. Afterward, the lumbar spinal cord of each animal was collected, included in cryopreservation medium and frozen in liquid-nitrogen-cold n-hexane with temperatures ranging from −30 to −35°C. The cryopreserved tissues were kept at −20°C until they were sectioned in cross sections of 12 μm using a cryostat (MICROM, model HM505E). The sections were performed in a series of 1:10 (120 μm apart). Five sections were placed per gelatinized slide, performing a total distance of about 500 μm of per slide. The slides were kept at −20°C until use for the study of the demyelination volume and for the immunofluorescence protocols.

### Assessment of demyelination volume

The slides were acclimatized at room temperature for 2–3 h and then washed in distilled water for 1 min followed by 1 min of dehydration in 70% alcohol. Then, the slides were incubated in Sudan Black solution for 20 min, washed three times in 70% alcohol and rehydrated in distilled water for 30 s. Finally, the slides were mounted using a 1:3 mixture of glycerol and phosphate buffer (PB) and kept at −20°C.

The stained slides were observed under a Leica DM 5500B light microscope. For quantification purposes, the first, third, and fifth cuts of each slide were photographed, making a scan of about 500 μm of lumbar spinal cord extension, using a high sensitivity camera (Nikon, DXM 1200F). To investigate the demyelination of the anterior, lateral, and posterior funiculi, images of the entire length of the spinal cord sections were obtained using a 10X objective and, by using Adobe Photoshop Elements 10 program, entire images of the spinal cord were assembled and quantified using IMAGEJ software (version 1.49v, National Institutes of Health, USA).

For quantification, the demyelinating lesions were calculated as the percentage of the total myelin area. To include a descriptive analysis of the lesions, the number of demyelinated areas were evaluated per photo per animal. Such analysis allowed a qualitative description of the regions of demyelination (motor and sensory) that is demonstrated in the Supplementary Material.

### Immunofluorescence protocols and quantification

The slides were acclimatized at room temperature for 15 min and then washed in PB (3 times of 5 min each). After incubation for 45 min with blocking solution containing 3% fetal bovine serum (BSA), the slides were incubated for 3 h with primary antibodies. Anti-Iba1 (*Ionized calcium binding adaptor molecule 1*; Rabbit, Wako 019-19741, 1:750) was used to evaluate the microglial reaction as well as the infiltration of macrophages derived from monocytes to the spinal cord microenvironment (21). Anti-GFAP (*glial fibrillary acidic protein;* Rabbit, Abcam AB7260, 1:1,500) was used to evaluate astrocytes reaction and anti-synaptophysin (Rabbit, Novusbio NBP2-25170, 1:1,000) to evaluate synaptic covering (23). Afterward, the slides were washed with PB and incubated for 45 min with the fluorescent secondary antibody (Cy3 Donkey Anti-Rabbit IgG, Jackson, 1:500). Finally, the slides were washed in PB, assembled using a 1:3 mixture of glycerol and PB and reserved at −20°C.

Immunolabeled slides were observed on a Leica DM 5500B fluorescence microscope coupled to a Leica DFC 345FX camera using the rhodamine (CY3) filter. For quantification purposes, the first, third, and fifth sections of each slide were photographed, covering a 500 μm depth of the lumbar spinal cord for each analysis, using a high sensitivity camera (Nikon, DXM 1200F). Images of ventral (Lamina IX), dorsal (Laminas I-II), and central canal regions were captured using a 20X objective for the sections labeled with anti-Iba1 and anti-GFAP. Images of the ventral region (Lamina IX) were captured using a 40X objective for the sections labeled with anti-synaptophysin using the “maximum projection” feature for adequate focusing. For the quantification, the integrated density of pixels (IDP) was performed using IMAGEJ software (version 1.49v, National Institutes of Health, USA). For Iba1 and GFAP, measurements were taken on the whole photos and for synaptophysin, the measurement was restricted in eight equidistant points of two neurons per photo (24-pixel circles). The mean IDP was calculated for each animal and then the mean of the ratios for each group ± standard error was established and normalized against the naive group.

### Statistical analysis

**Figure 1:**
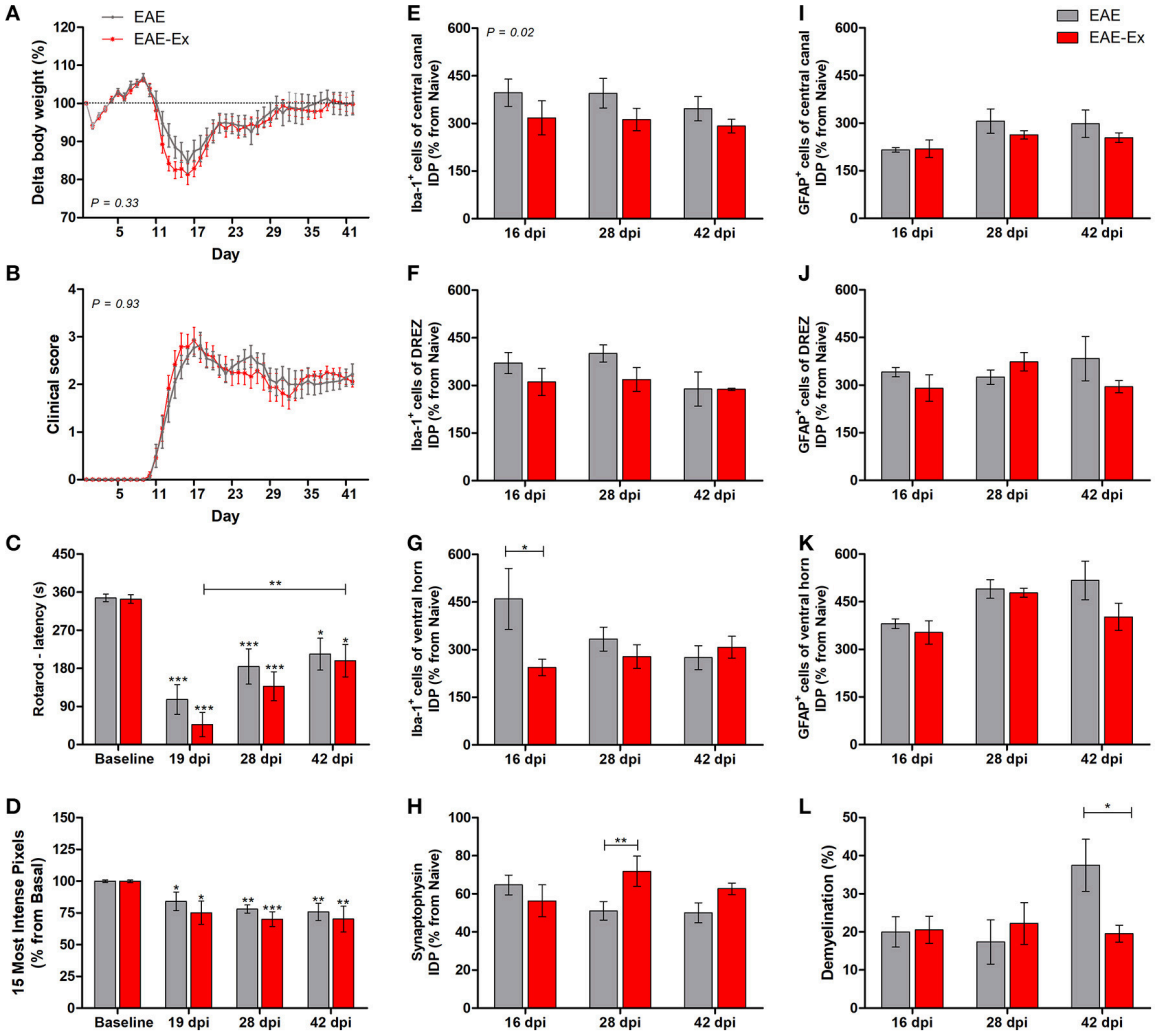
Effects of 6 weeks of treadmill exercise on EAE disease progression. **(A)** Delta body weight. **(B)** Clinical score presentation. **(C)** Rotarod motor test (latency to fall in seconds). **(D)** Intensity of the foot print. **(E-G)** IDP (integrated density of pixels) quantification of anti-Iba1 pictures from central canal **(E)**, dorsal root entry zone—DREZ **(F)**, and ventral horn **(G)**. **(H)** IDP quantification of anti-synaptophysin pictures from ventral horn. **(I–K)** IDP quantification of anti-GFAP pictures from central canal **(I)**, dorsal horn entry zone—DREZ **(J)**, and ventral horn **(K)**. **(L)** Percentage of demyelinated areas. *P < 0.05, ^**^P < 0.01, or ^***^P < 0.0001.

**Figure 2:**
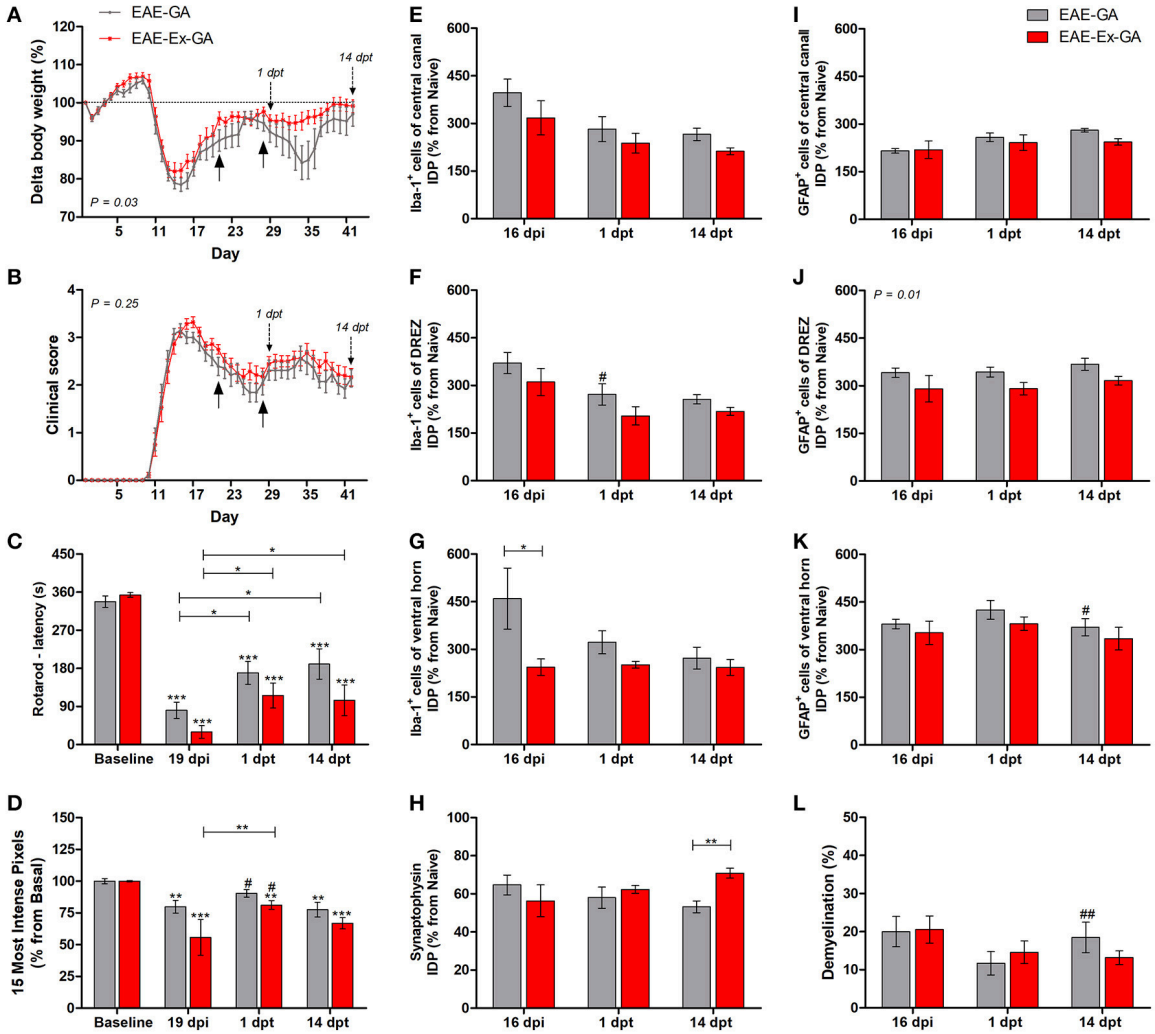
Effects of Glatiramer Acetate (GA) treatment after 21 dpi in EAE mice that were submitted to 6 weeks of treadmill exercise. **(A)** Delta body weight (*P* < 0.05). **(B)** Clinical score presentation. The arrows below the curves indicate the start and the end of GA treatment and the arrows over the curves indicate 1 and 14 days after the end of GA treatment. **(C)** Rotarod motor test (latency to fall in seconds). **(D)** Intensity of the foot print. **(E–G)** IDP quantification of anti-Iba1 pictures from central canal **(E)**, dorsal root entry zone—DREZ **(F)**, and ventral horn **(G)**. **(H)** IDP quantification of anti-synaptophysin pictures from ventral horn. **(I–K)** IDP quantification of anti-GFAP pictures from central canal **(i)**, dorsal horn entry zone - DREZ **(J)**, and ventral horn **(K)**. **(L)** Percentage of demyelinated areas. ^*^P < 0.05, ^**^P < 0.01, or ^***^P < 0.0001. ^#^*P* < 0.05 and ^*##*^*P* < 0.01, respectively, between GA treated and untreated animals.

**Figure 3:**
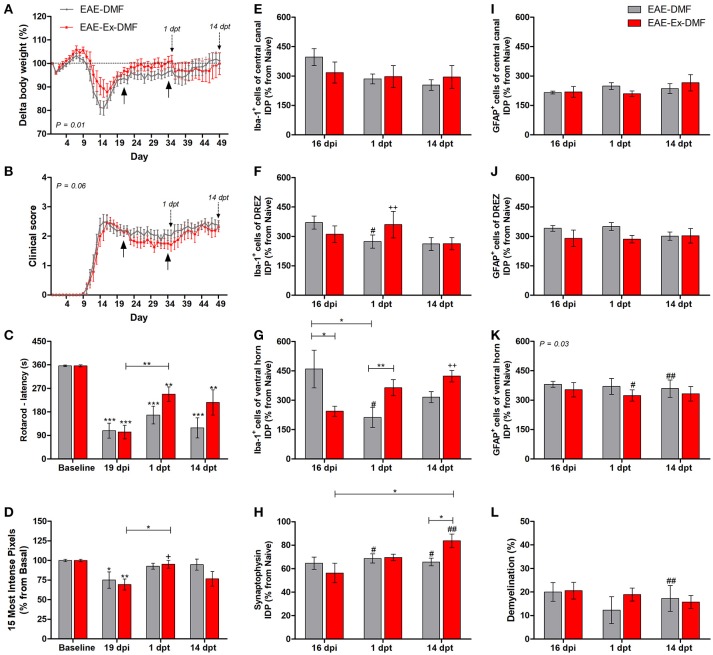
Effects of Dimethyl Fumarate (DMF) treatment after 21 dpi in EAE mice that were submitted to 6 weeks of treadmill exercise. **(A)** Delta body weight (*P* < 0.05). **(B)** Clinical score presentation. The arrows below the curves indicate the start and the end of DMF treatment and the arrows over the curves indicate 1 and 14 days after the end of DMF treatment. **(C)** Rotarod motor test (latency to fall in seconds). **(D)** Intensity of the foot print. **(E–G)** IDP quantification of anti-Iba1 pictures from central canal **(E)**, dorsal root entry zone–DREZ **(F)**, and ventral horn **(G)**. **(H)** IDP quantification of anti-synaptophysin pictures from ventral horn. **(I–K)** IDP quantification of anti-GFAP pictures from central canal **(I)**, dorsal horn entry zone—DREZ **(J)**, and ventral horn **(K)**. **(L)** Percentage of demyelinated areas. ^*^P < 0.05, ^**^P < 0.01, or ^***^P < 0.0001. ^#^*P* < 0.05 and ^*##*^*P* < 0.01, respectively, for the comparison between DMF treated and untreated animals. ^+^*P* < 0.05 and ^++^*P* < 0.01, respectively, for the comparison between DMF treated and the other two groups.

Statistical analyses were performed with GraphPad Prisma 4.0 software and data are shown as mean ± standard error. Firstly, all data were subject to a two-way analysis of variance (ANOVA) with *Bonferroni* post-test to investigate the percentage of variation caused by the exercise during the progression of disease with or without the drug treatments. From these analyses, the differences between no-exercise and exercise are demonstrated on Figures 1G,H,L, 2G,H, 3G,H by the capped lines. Secondly, the whole time point curves where compared by Mann-Whitney test between no-exercise and exercised groups of mice. The *P*-values demonstrated at the corner of the Figures 1A,B,E, 2B,J, 3A,B,K are indicative of this analysis. Thirdly, one-way ANOVA with Newman-Keuls multiple comparison tests was used to analyze intragroup variation. These are the comparisons made between basal and disease time points or even between the different disease time points (days post induction or days post-treatment). Finally, the sharp and the plus signs indicate the intergroup differences that were obtained by two-way analysis of variance (ANOVA) between all studied groups at 28 dpi/1 dpt and 42/14 dpt. The two-way analysis of variance was followed by Bonferroni post-test and the sharp symbol on Figures 2D,F,K,L, 3G,H,K,L indicate differences from untreated animals while the plus signs on Figures 3D,F,G indicate difference between DMF treated animals and the other two set of animals (untreated and GA treated). In all cases, the differences between groups were considered significant when *P*-values were minor than 0.05.

## Results

### Prior treadmill exercise modified histopathological hallmarks

In the first set of results, the global effect of the protocol of exercise used herein on behavioral and histopathological parameters of EAE mice was observed. For the longitudinal behavioral analyses, a group of 12 animals per group was used and, for the histopathological analyses, a group of 4–6 animals per group per time point was used. No statistical differences were observed between no-exercise and exercised groups regarding the body weight evolution (Figure 1A), clinical score presentation (Figure 1B), latency to fall from rotarod apparatus (Figure 1C), and the pressure of the hind limb paw print (Figure 1D). Importantly, a clinical attenuation effect by exercise was observed once such group of animals did not present the second clinical relapse (Figure 1B). The delayed and attenuated second relapse group may be associated to the reduced integrated density of pixels (IDP) of Iba-1 immunofluorescence, which is related to microglial reactivity and infiltration of macrophages, for the exercised animals in comparison to the no-exercise group. This observation was made at peak disease in the ventral horn (*P* < 0.05 for 16 dpi on Figure 1G) and throughout the complete studied period for the central canal region (*P* < 0.05 on Figure 1E). On the other hand, exercised EAE animals presented an increased synaptic density coverage around the motoneurons at 28 dpi (*P* < 0.05 for 28 dpi on Figure 1H; red arrowheads on panel B of **Figure 6**). These results were followed by a reduced progression of the white matter demyelination at 42 dpi (*P* < 0.05 for 42 dpi on Figure 1L). For the reduced number of areas of demyelination, see also the Supplementary Figure 1. No differences for GFAP immunoreactivity were observed between the animals that underwent no exercise and those with EAE that were exercised (Figures 1L,J,K).

**Figure 4:**
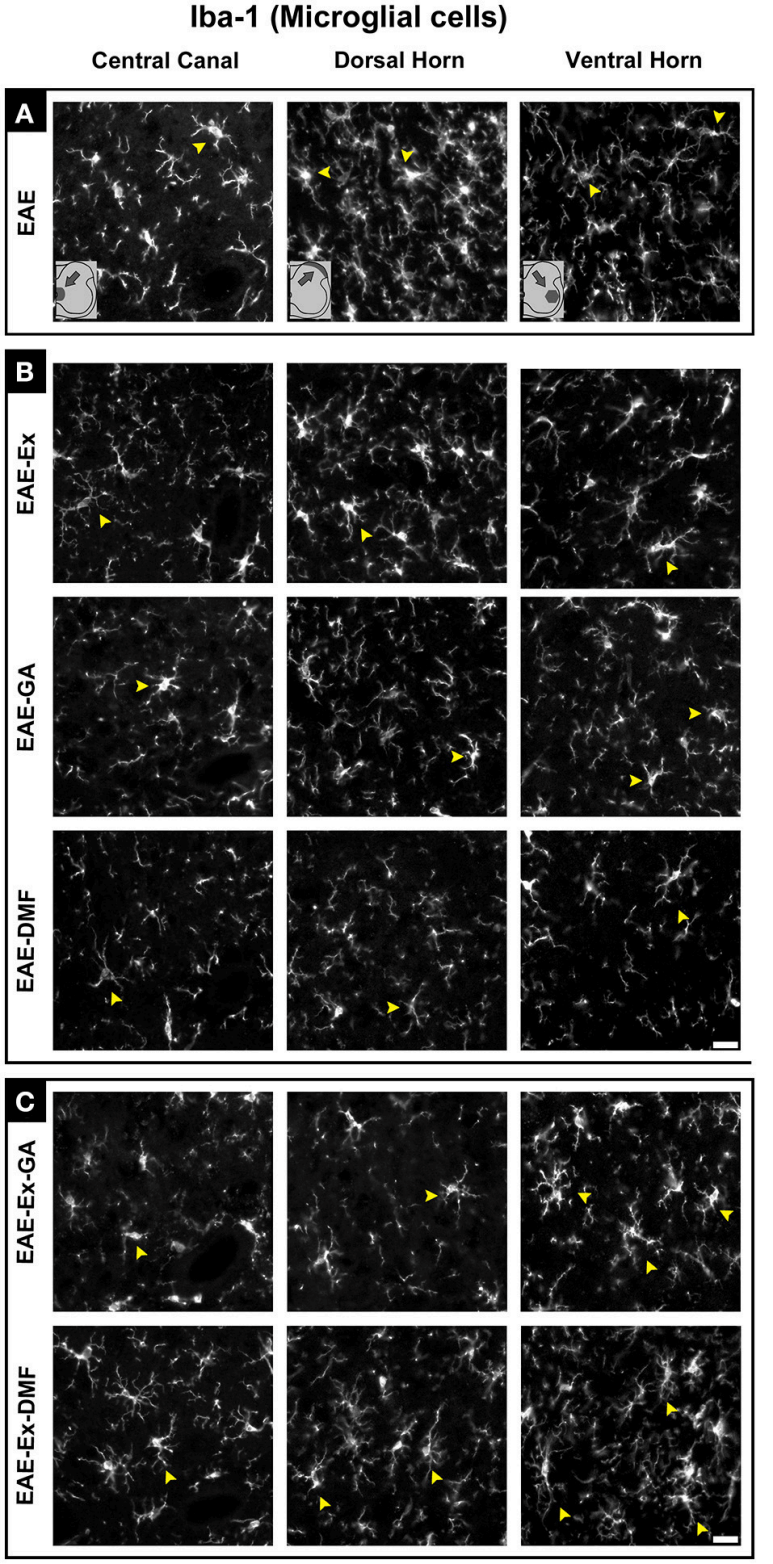
Representative pictures of sections immunolabeled with anti-Iba1 for microglial/macrophage cells. Although all the time points have been quantified, an illustrative demonstration of iba-1 immunofluorescence at different time points is presented in this figure (detailed information on the text). The pictures are organized in three columns as central canal, dorsal horn, and ventral horn. Besides, they have been divided into three panels according to the experimental design: **(A)** for EAE animals; **(B)** for exercised and isolated drug-treated animals and; **(C)** for the associations (exercise and drug treatment). Some of the positive branched stained cells are indicated by the yellow arrowheads. Note the more branched morphology of all EAE-Ex-DMF pictures (compare the arrows narrows all over the pi). Scale bar: 10 μm.

**Figure 5:**
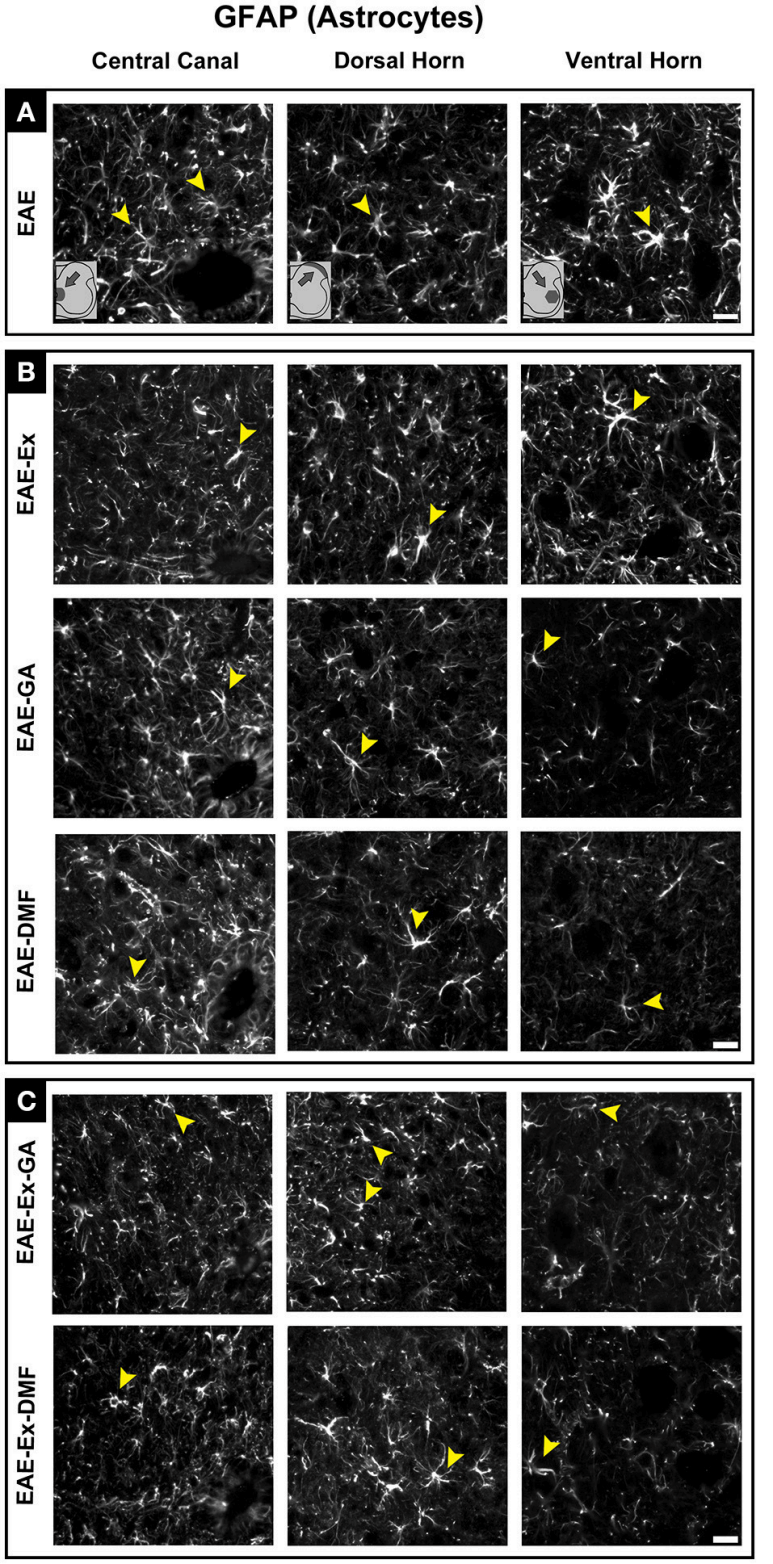
Representative pictures of sections immunolabeled with anti-GFAP for astrocytes. Although all the time points have been quantified, illustrative GFAP immunofluorescence at different time points is presented (detailed information in the text). The pictures are organized in three columns as central canal, dorsal horn, and ventral horn. Besides, they have been divided into three panels according to the experimental design: **(A)** for EAE animals; **(B)** for exercised and isolated drug-treated animals and; **(C)** for the associations (exercise and drug treatment). Some of the positive branched stained cells are indicated by the yellow arrowheads. Observe the GFAP immunofluorescence around the motoneurons on ventral horn pictures. Scale bar: 10 μm.

**Figure 6:**
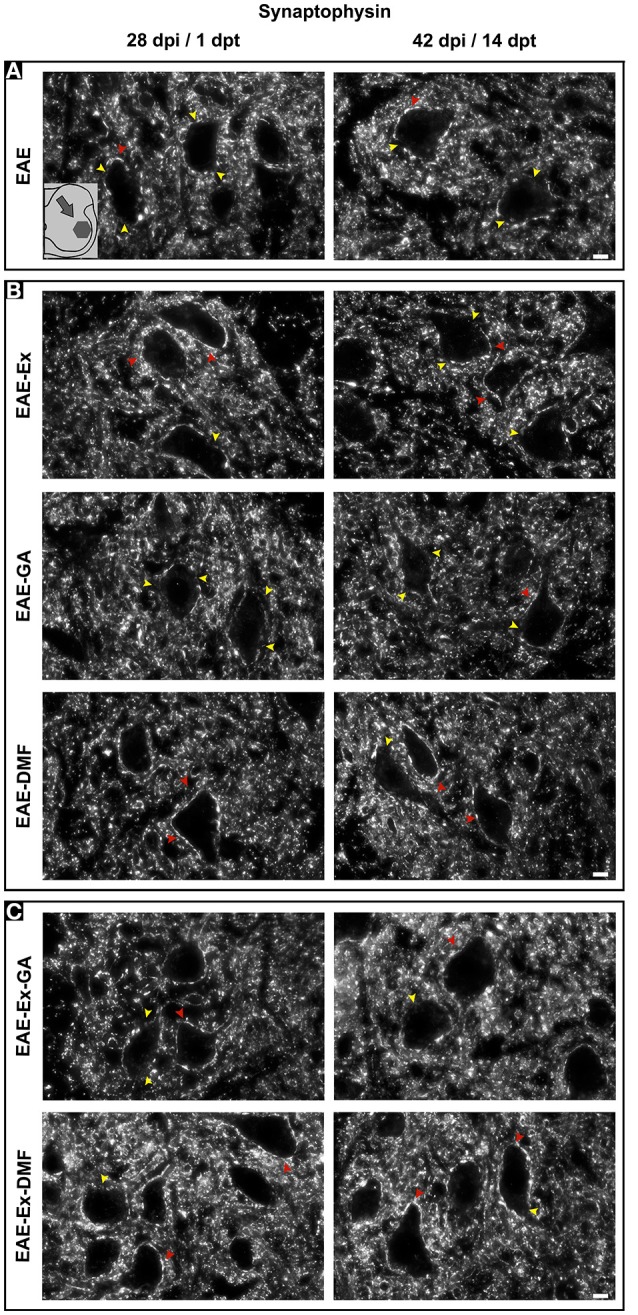
Representative pictures of sections immunolabeled with anti-synaptophysin. The pictures are organized in two columns as 28 dpi (EAE and EAE-Ex groups)/1 dpt (EAE-GA. Besides, they have been divided into three panels according to the experimental design: **(A)** for EAE animals; **(B)** for exercised and isolated drug-treated animals and; **(C)** for the associations (exercise and drug treatment). Note the areas with positive immunolabeling around motoneurons (red arrowheads) and the areas of synaptic damage (yellow arrowheads). Also note increased areas of positive immunolabeling of exercised animals. Scale bar: 10 μm.

**Figure 7:**
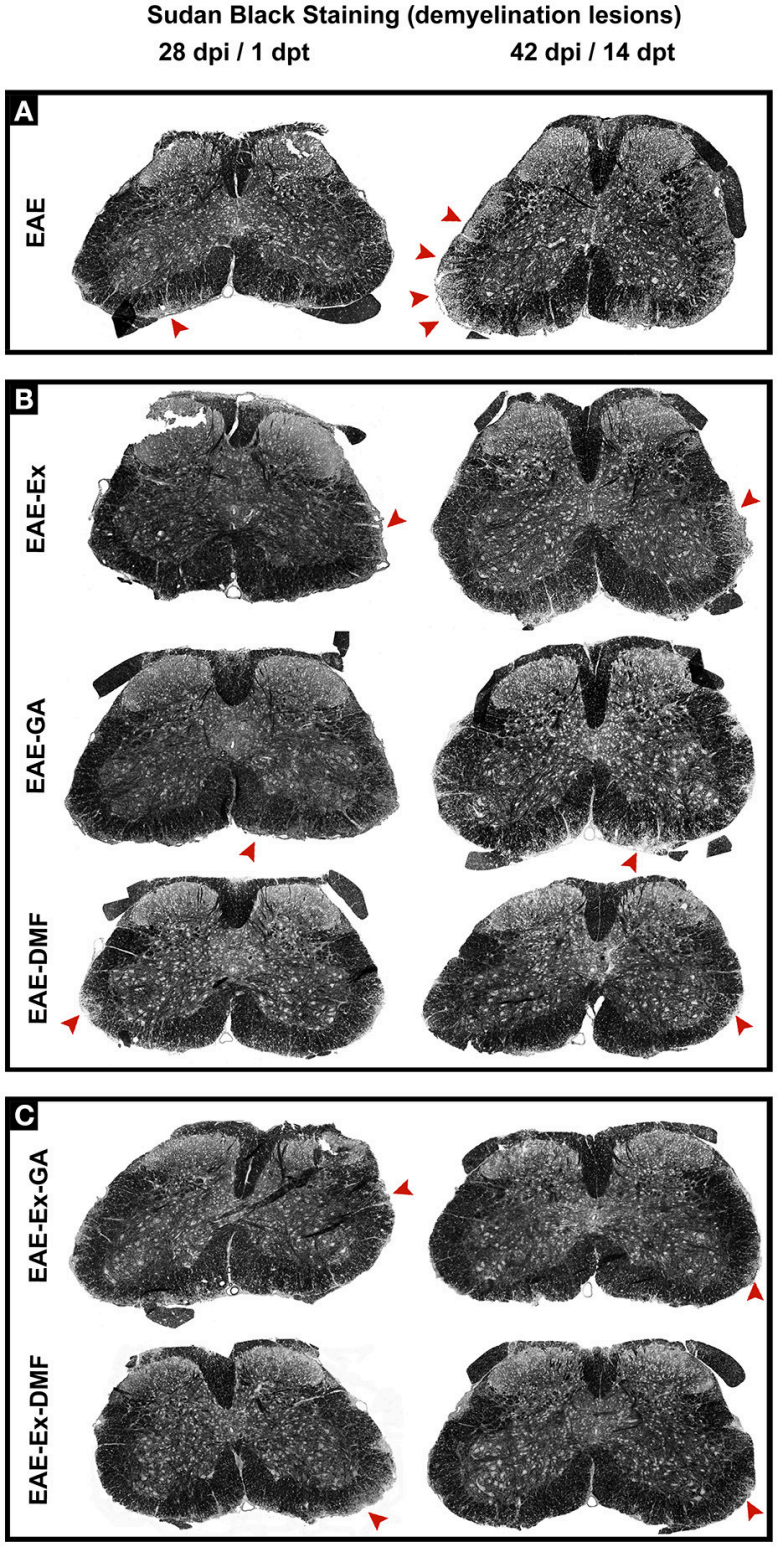
Representative pictures of sections stained with Sudan black and used to measurement of demyelinating lesions. The pictures are organized in two columns as 28 dpi Besides, they have been divided into three panels according to the experimental design: **(A)** for EAE animals; **(B)** for exercised and isolated drug-treated animals and; **(C)** for the associations (exercise and drug treatment). Note the areas of demyelination pointed with the red arrowheads.

Although all-time points have been quantified, illustrative Iba-1 immunofluorescence at different time points is presented in Figure 4. Therefore, the pictures from central canal area are from animals euthanized at 42 dpi while the pictures from dorsal and ventral horn are from animals euthanized at 28 dpi (compare panel A to the first line of panel B on Figure 4). Similarly, representative pictures of sections immunolabeled with anti-GFAP which is related to astrocyte reactivity are presented in Figure 5 at different time points. The pictures from the central canal and dorsal horn areas are from animals euthanized at 28 dpi while the pictures from ventral horn are from animals euthanized at 42 dpi. Figure 6 illustrates the motoneurons from the ventral horn area that have their presynaptic inputs immunolabeled with anti-synaptophysin. In such case, only the peak disease (16 dpi) time point is not demonstrated. On EAE motoneurons (panel A) it is possible to observe that the immunolabeling of synaptophysin presents some gaps, i.e., it is not continuous, that is suggestive of synaptic loss/pruning (yellow arrowheads). On the other hand, it is noticeable the increased synaptophysin immunolabeling (reduced gaps) around motoneurons from the exercised animals' pictures in comparison to the no-exercise group at 28 dpi (red arrowheads). Finally, Figure 7 illustrates pictures of sections stained with Sudan black that was used for the measurement of demyelinating lesions. Observe the red arrowheads to identify the demyelinated areas. Noteworthy, Figures 4–7 also present illustrative pictures from drug-treated animals (panels B and C).

### GA treatment promoted modification of clinical, behavioral, and histopathological hallmarks of the disease

This second group of data are related to the no-exercise and exercised EAE mice which have received glatiramer acetate from 21 to 27 dpi, together with the EAE-GA (no-exercise) and EAE-Ex-GA (exercised) groups. Therefore, the associated effect from the protocol of exercise used herein and the treatment with GA on behavioral and histopathological parameters of EAE mice was observed. As described in the Methods section, 12–14 animals/group was used for the behavioral analyses and five animals per group per time point were used for the histopathological analyses. From this set of experiments, the interruption of the treatment negatively affected the body weight curve only for the no-exercise animals, suggesting a protective effect of the exercise on feeding behavior (Figure 1A; *P* < 0.05 between EAE-GA and EAE-Ex-GA). However, both groups of animals presented the second clinical relapse just after the end of the treatment (Figure 2B) which was followed by an intragroup recovery of the latency to fall on rotarod (Figure 2C) and an intergroup increased pressure of the hind limb paw print (Figure 2D vs. Figure 1D; represented by the sharp symbol on the 1 dpt bars).

The asterisk signs over the bars of Figures 2C,D represent intragroup variation between basal time point and 19 dpi and they were obtained by one-way ANOVA with Newman-Keuls multiple comparison tests. This analysis also shows differences between 19 dpi and 1 dpt and 14 dpt (capped lines). Noteworthy, there was no difference between basal and 1 dpt for EAE-GA group on Figure 2D. The sharp symbol on the 1 dpt bars of Figure 2D was obtained by two-way analysis of variance (ANOVA) with Bonferroni post-test and indicates the intergroup variation of the hind limb paw print. In this case, the sharp symbols indicate a difference between GA treated and no-treated animals (Figure 2D vs. Figure 1D at similar time points). Therefore, Figures 2C,D present data that are suggestive of an effect from GA on motor recovery and paw pressure improvement.

Noteworthy, although both GA treated groups presented higher values of paw print pressure than the untreated counterparts at 1 dpt, a differential glial response on the dorsal horn region was observed. A diminished Iba-1 immunoreactivity was observed for the EAE-GA group compared to EAE group (sharp symbol on Figure 2F vs. Figure 1F and Figure 4). On the other hand, a global attenuation on astrocytic immunoreactivity was observed for the EAE-Ex-GA group in comparison with EAE-GA group (Figures 2J, 5). Since the intensity of the pressure applied during a paw stance have been associated with development of chronic mechanical allodynia, these data may suggest a positive effect of GA on the neuropathic pain or allodynia via differential glial mechanisms between no-exercise and exercised EAE mice.

In the same way, it seems that GA treatment accounted for a decreased astrocyte immunoreactivity in the ventral horn at 14 dpt (sharp symbol on Figure 2K vs. Figure 1K and Figure 5) and attenuated demyelination lesion at 14 dpt (sharp symbols on Figure 1L vs. Figure 2L and Figure 7) only for the no-exercise animals. However, no differences for both Iba-1 and GFAP immunoreactivity were observed between animals that underwent no exercise and those with EAE that were exercised at the central canal region (Figures 2E,I). Lately, there was a recovery of the synaptic density coverage around the motoneurons at 14 dpt for the EAE-Ex-GA in comparison to the EAE-GA group (Figures 1H, 6), suggesting that the GA treatment delayed the synaptic alterations for the no-exercise animals.

### DMF promoted pronounced modification of clinical, behavioral, and histopathological hallmarks of the disease

The third and last set of comparisons (Figure 3) brings information related to the no-exercise and exercised EAE mice that were treated with DMF (EAE-DMF and EAE-Ex-DMF groups). With these experiments, the associated effect from the protocol of exercise and the treatment with DMF on behavioral and histopathological parameters of EAE mice was observed. As described in the Methods section, 14 no-exercise and 12 exercised animals were used in the behavioral analyses and five animals per group per time point was used for the histopathological analyses. Firstly, DMF treatment delayed the second relapse to the first-day post-treatment (35 dpi) in both groups of animals which can be observed by both body weight (Figure 3A) and clinical score (Figure 3B) curves. However, there was a difference (*P* < 0.05) between no-exercise and exercised mice treated with DMF regarding the body weight (Figure 3A) and clinical results (Figure 3B). Therefore, these data suggest a global positive effect from the association (exercise plus DMF treatment), especially during the drug treatment period.

EAE-Ex-DMF animals presented intragroup recovery of both motor and sensitive outcomes represented, respectively, by the latency to fall from the rotarod apparatus (Figure 3C) and by the pressure of the hind limb paw print (Figure 3D). In this regard, the asterisk signs over the bars of Figures 3C,D represent intragroup variation and they were obtained by one-way ANOVA with Newman-Keuls multiple comparison tests and they indicate differences from basal time point. The same analysis showed a statistical difference between 19 dpi and 1 dpt for exercised group (EAE-Ex-DMF) on both Figures 3C,D. Noteworthy, observe the lack of difference between basal and both dpt time points (1 and 14) for both groups (EAE-DMF and EAE-Ex-DMF) on Figure 3D. The pressure of the hind limb paw print at 1 dpt for the EAE-Ex-DMF was also higher than the other two exercised groups (vs. EAE-Ex and vs. EAE-Ex-GA). This result is represented by the plus sign and it was obtained by two-way analysis of variance (ANOVA) with Bonferroni post-test.

Interestingly, these beneficial behavioral results from the exercised DMF animals were paralleled by increased Iba-1 immunolabeling. Thus, Figure 3F show increased Iba-1 immunolabeling on the dorsal horn at 1 dpt and Figure 3G show increased Iba-1 immunolabeling on the ventral horn at 14 dpt. The differences are demonstrated by double plus sign on the red bars and they indicate difference from EAE-Ex and EAE-Ex-GA groups. In fact, note that all plus signs on Figures 3D,F,G occur over red bars (exercised animals). Noteworthy, on Figure 3G is possible to observe that the Iba-1 immunolabeling was significantly higher for the exercised group in comparison to the no exercised group at 1 dpt (capped line). No-exercise animals treated with DMF showed decreased Iba-1 (microglia/macrophage) immunolabeling at 1 dpt on both dorsal (Figure 3F) and ventral (Figure 3G) horns in comparison to untreated animals (represented by the sharp symbols).

Albeit this differential microglial/macrophage response between the two DMF groups, both demonstrated increased synaptic density of covering around the motoneurons at 14 dpt in comparison to the untreated animals (Figure 3H, represented by sharp symbols on gray and red bars). Nevertheless, the preservation synaptophysin immunolabeling was more pronounced for the EAE-Ex-DMF (*P* < 0.05 vs. EAE-DMF group; Figures 3H, 6), suggesting a synergistic response of exercise and DMF treatment.

## Discussion

The main contribution of the present study is providing evidence of positive interaction between regular and prior physical exercise and the use of disease-modifying therapies (DMTs) during the progression of an animal model for multiple sclerosis (MS). In a perspective of translational studies, our findings are aligned with the concept that an active lifestyle person which is eligible to take an MS drug treatment after the first relapse of the disease would be more responsive to treatment. Noteworthy, the characteristic relapsing model in C57BL/6J mice has been demonstrated before, suggesting that the amount or even the source of the antigen, as well as the adjuvant used to induce EAE may impact the clinical course of the disease (22, 23). This allows to investigate the potential of different treatments during relapses. Therefore, although our exercise protocol only promoted slight alterations in the second clinical relapse, that was enough to modify the clinical response and histopathological hallmarks after the DMTs introduction.

Forced treadmill exercise paradigm has been used on EAE animals with conflicting results regarding the clinical score. Therefore, worsening of symptoms (24), no effect on disability (25–27), delayed day of peak disease (28) or of onset of disease (29) and even a global amelioration of clinical signs (30, 31) has been demonstrated on EAE animals that have been exercised on treadmill. Nevertheless, different EAE models, as well as different volume, intensity and duration of exercise, may affect final conclusions. Albeit the conflicting clinical results, some beneficial morphological and/or molecular effects from exercise on EAE animals such as preservation and modification of skeletal muscle parameters (24, 28) and increased neurotrophic factor expression (24, 27) were observed when the clinical score was not ameliorated or even worsened. Therefore, our results suggest that, even though the exercise protocol used herein did not alter the development of the disease, it may promote some important histopathological modifications which could be optimized by a DMT strategy.

Body weight was significantly improved by the association in both cases, suggesting that the systemic metabolic alterations prompted by exercise may have acted in synergy with the DMTs. It has been demonstrated that the levels of leptin are increased before the EAE clinical presentation and that these results are associated with disease severity, food-intake inhibition, and body-weight loss (32) while regular physical exercise is classically recognized to reduce the circulating leptin levels (33). It is important to note that leptin hormone may also regulate microglial function and leukocyte recruitment in neuroinflammation creating an important link between energy balance and immune function (34, 35). In this regard, future studies are necessary to evaluate the relationship between leptin and inflammation in EAE mice.

The fact that exercised and treated animals presented higher values of paw pressure at 1 day after treatments (1 dpt) were not related with such weight preservation but rather with a protection against inflammation. This is reinforced by the fact that we demonstrated herein a reduced intensity of the paw prints in EAE mice, possibly related to mechanical allodynia as it has been suggested before (20, 36). Compared with EAE animals at a similar time point, isolated GA treatment has decreased Iba-1 immunolabeling at 1 dpt in the dorsal horn. Similarly, systemic administration of GA is capable of declining neuropathic hypersensitivity and inhibiting microglial response in the spinal cord dorsal horn of animals submitted to peripheral nerve injury (37). The same authors also suggest that Th1/Th2 balance within the spinal cord may be a potential therapeutic strategy for neuropathic pain. That rationale would also be applicable to the EAE-DMF group which has demonstrated a similar decreased microglia/macrophage response in the dorsal horn. Taken together, our data suggest that both DMTs used herein have the potential to reduce inflammatory profiles that may contribute to the attenuation of neuropathic pain in the EAE model.

Exercised animals demonstrated no additional effect of GA treatment on dorsal horn microglia/macrophage data but a global and significant attenuation of astrocyte immunoreactivity. With the accumulation of inflammatory mononuclear cells in the CNS of EAE animals, astrocytes start to produce and release CCL2 which is involved in the amplification of the immune response and progression of the disease (38). In the dorsal horn, CCL2 released by astrocytes has also been associated with neuropathic pain condition once its receptor CCR2 occurs in activated microglia but also in dorsal horn neurons where it may have a role in the enhancement of the glutamatergic synaptic transmission (36). Therefore, the synergistic effect of exercise and GA on decreasing GFAP marker at the dorsal horn may be a mechanism for the increased paw pressure intensity of these animals. Instead, GA treatment on exercised EAE animals did not show any effect on ventral horn glial cells and demonstrated only a significant increased synaptic density cover at 14 dpt. Once this result was similar to what has been observed with untreated animals at 28 dpi, it may indicate that GA alone delays synaptic damage rather than having a synergic effect with exercise.

Interestingly, the best result of paw pressure was associated with an increased Iba-1 immunoreactivity in the dorsal horn at 1 dpt (EAE-Ex-DMF group). Some evidence suggests a neuroprotective role of microglia in adult CNS such as clearance of debris that helps to reduce inflammation, maintenance of synapse homeostasis and production of neurotrophic as well as neuromodulatory factors (39, 40). Noteworthy, it has been suggested that exercise may promote neuroprotection by modulating circulating macrophages that infiltrate the CNS and then regulate neurotoxic microglia (41). Besides, DMF has been suggested to promote stimulation of some cellular pathways in activated microglia that could be associated with the preservation of neurons (42). Therefore, it may be possible that exercise and DMF have acted in synergy, optimizing the microglial/macrophage response toward a neuroprotective profile. In this way, further studies are necessary to better clarify such hypothesis.

Importantly, the increased Iba-1 immunoreactivity was also observed in the ventral horn at 1 and 14 dpt for these animals. In addition, this result was paralleled with ventral horn decreased astrocytic reactivity at 1 dpt and increased synaptic density cover at 14 dpt. Synaptic density cover was measured by synaptophysin immunoreactivity which is a synaptic protein localized on the membrane of vesicles of presynaptic terminals and illustrates the inputs to the alpha motor neurons, allowing an inference of axonal damage and a general analysis of spinal cord circuit integrity (21). Accordingly, previous studies with EAE animals have demonstrated synaptic pathology with reduced immunoreactivity for synaptophysin at peak disease while its upregulation has been associated with clinical recovery and neuroprotection (21, 43). Of note, the increments on synaptophysin immunoreactivity are not always associated with a proportional reduction on Iba-1 immunoreactivity at chronic disease (21, 44). Notably, the results with isolated DMF treatment on the ventral horn were similar to isolated exercise on the progression of the disease which was not observed with isolated GA treatment.

Overall, the present study provides some information related to the interaction between physical exercise and DMTs that should be further investigated. Noteworthy, isolated physical exercise and the two DMTs used herein have decreased the demyelinating lesions, suggesting an overall effect on the white matter. However, exercised-GA treated animals demonstrated decreased astrocytic response in the spinal dorsal horn with an improvement in the paw print pressure. On the other hand, exercised-DMF treated animals showed an increased microglial/macrophage response on both ventral and dorsal horn that were associated with clinical improvement and synaptic motoneuron inputs density. These results suggest a positive interaction between immunomodulation and exercise, resulting in synaptic stability and circuitry preservation. Therefore, the present data reinforce that prior regular exercise can modify the effects of pharmacological treatment administered after the first relapse in a murine model for MS.

## Availability of data and materials

The datasets supporting the conclusions of this article are included in the present submission.

## Author contributions

DB and AdO contributed to conception and design and wrote the paper. DB contributed to acquisition, analysis, and editing of data. AdO provided the supervision. All authors read and approved the final manuscript.

## Conflict of interest statement

The authors declare that the research was conducted in the absence of any commercial or financial relationships that could be construed as a potential conflict of interest.

## Supplementary material

The Supplementary Material for this article can be found online at: https://www.frontiersin.org/articles/10.3389/fneur.2018.00950/full#supplementary-material

Supplementary Figure 1 Number of demyelinated areas (motor and sensory) evaluated per photo/animal. Motor descending pathways included pyramidal (corticospinal) and extrapyramidal tracts (rubrospinal, reticulospinal, olivospinal, and vestibulospinal) while sensory ascending pathways included dorsal column medial lemniscus system (gracile fasciculus), spinocerebellar tracts (posterior and anterior), and anterolateral system.

## Abbreviations

ANOVA: analysis of variance
CEMIB/UNICAMP: Multidisciplinary Center for Biological Research of University of Campinas
CEUA/UNICAMP: Ethics Committee on Animal Experimentation of University of Campinas
CFA: complete Freund's adjuvant
CNS: central nervous system
CONCEA: National Council of Animal Experimentation
dpi: days post induction
DMF: dimethyl fumarate
DMTs: disease modifying therapies
dpt: days post-treatment
EAE: experimental autoimmune encephalomyelitis
EAE-DMF: EAE animals treated with dimethyl fumarate
EAE-Ex: EAE animals submitted to prior regular exercise
EAE-Ex-DMF: EAE animals submitted to prior regular exercise and treated with dimethyl fumarate
EAE-Ex-GA: EAE animals submitted to prior regular exercise and treated with glatiramer acetate
EAE-GA: EAE animals treated with glatiramer acetate
GA: Glatiramer acetate
GFAP: glial fibrillary acidic protein
Iba1: Ionized calcium binding adaptor molecule 1
MOG: myelin oligodendrocyte glycoprotein
MRI: magnetic resonance imaging
MS: multiple sclerosis.
