# Supplementary figures and images for: Regular Exercise Modifies Histopathological Outcomes of Pharmacological Treatment in Experimental Autoimmune Encephalomyelitis
Source: Front Neurol. 2018 Nov 20;9:950. doi: 10.3389/fneur.2018.00950 (PMC6256135; doi:10.3389/fneur.2018.00950)

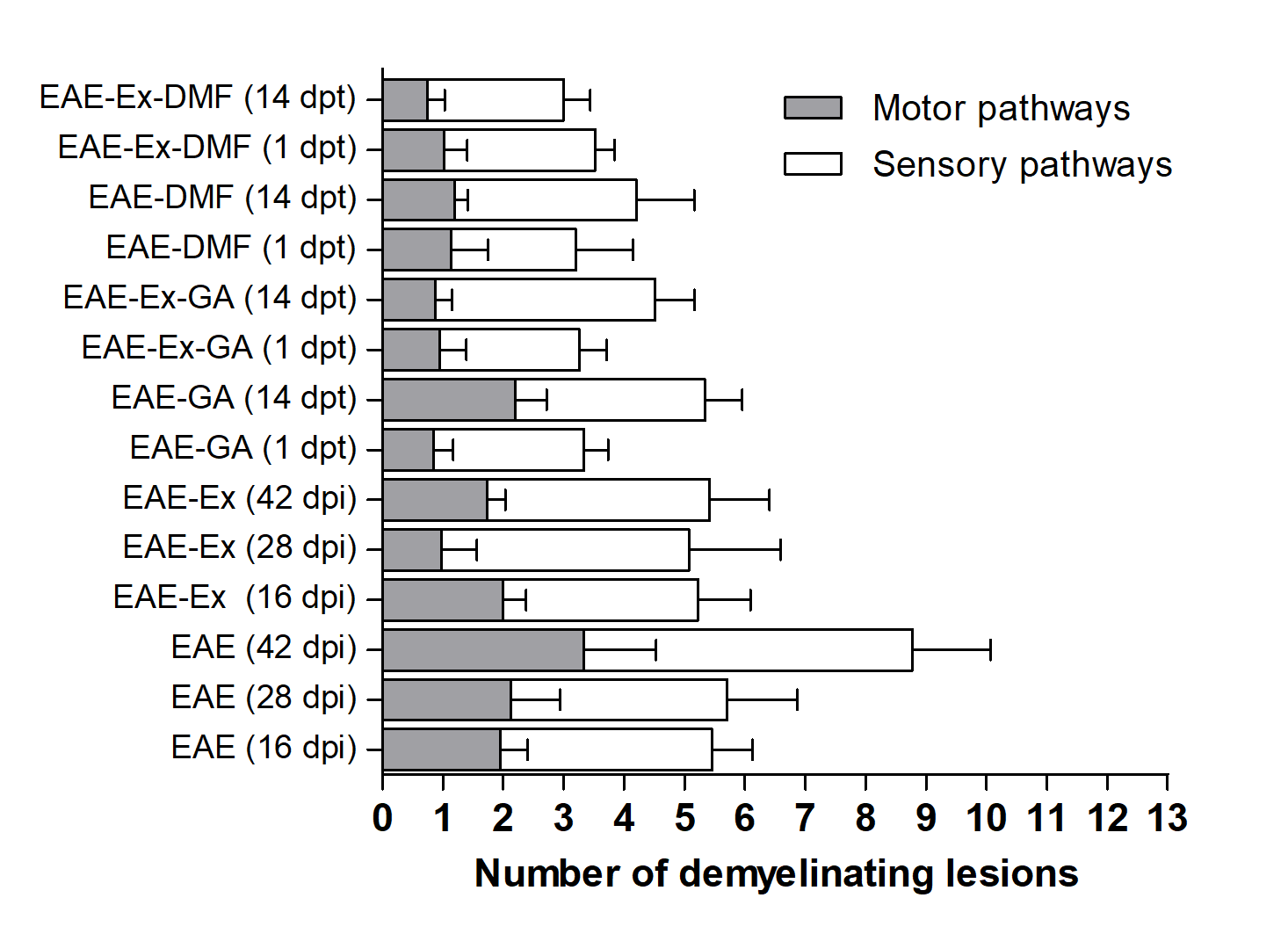

Supplement: Supplementary Figure 1 — Number of demyelinated areas (motor and sensory) evaluated per photo/animal. Motor descending pathways included pyramidal (corticospinal) and extrapyramidal tracts (rubrospinal, reticulospinal, olivospinal, and vestibulospinal) while sensory ascending pathways included dorsal column medial lemniscus system (gracile fasciculus), spinocerebellar tracts (posterior and anterior), and anterolateral system. [file Image_1.TIF]
